# Synergetic signal amplification of multi-walled carbon nanotubes-Fe_3_O_4_ hybrid and trimethyloctadecylammonium bromide as a highly sensitive detection platform for tetrabromobisphenol A

**DOI:** 10.1038/srep38000

**Published:** 2016-11-29

**Authors:** Feng Zhou, Yue Wang, Wei Wu, Tao Jing, Surong Mei, Yikai Zhou

**Affiliations:** 1 State Key Laboratory of Environment Health (Incubation), Key Laboratory of Environment and Health, Ministry of Education, Key Laboratory of Environment and Health (Wuhan), Ministry of Environmental Protection, School of Public Health, Tongji Medical College, Huazhong University of Science and Technology, #13 Hangkong Road, Wuhan, Hubei, China; 2 School of Laboratory Medicine, Hubei University of Chinese Medicine, #1 Hangjia Lake West Road, Wuhan, Hubei 430030, China

## Abstract

In this work, we fabricated an electrochemical sensor based on trimethyloctadecylammonium bromide and multi-walled carbon nanotubes-Fe_3_O_4_ hybrid (TOAB/MWCNTs-Fe_3_O_4_) for sensitive detection of tetrabromobisphenol A (TBBPA). The nanocomposite was characterized by X-ray diffraction (XRD), transmission electron microscopy (TEM) and Fourier transform infrared spectroscopy (FT-IR) techniques. The electrochemical behaviors of TBBPA on TOAB/MWCNTs-Fe_3_O_4_ composite film modified glassy carbon electrode (GCE) were investigated by cyclic voltammetry (CV), differential pulse voltammetry (DPV) and electrochemical impedance spectroscopy (EIS) method. The experimental results indicated that the incorporation of MWCNTs-Fe_3_O_4_ with TOAB greatly enhanced the electrochemical response of TBBPA. This fabricated sensor displayed excellent analytical performance for TBBPA detection over a range from 3.0 nM to 1000.0 nM with a detection limit of 0.73 nM (S/N = 3). Moreover, the proposed electrochemical sensor exhibited good reproducibility and stability, and could be successfully applied to detect TBBPA in water samples with satisfactory results.

Brominated flame retardants (BFRs) are present in many aspects of our lives. They are chemicals widely incorporated as additives into materials, such as plastic, textiles and electronic products, to prevent or slow down the spread of fire and make materials self-extinguishing from fires^1^,^2^. Tetrabromobisphenol A (TBBPA) is the most widely used BFRs, which covers around 60% of the total BFRs market^3^. The total global market demand for TBBPA amounted to over 170,000 tons in 2004^4^, and it is likely that the number has continued to increase in recent years.

A global concern was raised on TPPBA during past decades. The TPPBA could be detected extensively in the environment, as well as in bodies of human^5^,^6^,^7^,^8^,^9^,^10^,^11^,^12^. With the structural resemblance to the thyroid hormone, thyroxin, TBBPA can bind to human transthyretin and disrupt thyroid hormone functions^13^,^14^,^15^. Moreover, TBBPA was reported for its reproductive toxicity^16^, immunotoxicity^17^, neurotoxicity^18^,^19^. In a recent study, TBBPA-mediated uterine cancer has been shown in rodents exposed under laboratory conditions^20^. Therefore, with the view to its toxic effects on the human health, improving rapid and reliable methods for TBBPA determination in the field of environment monitoring and contaminants controlling is urgent.

Up to now, various analytical techniques have been developed for the determination of TBBPA, which includes high-performance liquid chromatography^21^, liquid chromatography tandem mass spectrometry^22^,^23^, gas chromatography-mass spectrometry^24^,^25^, surface-enhanced Raman scattering spectroscopy^26^ and immunosensor^27^,^28^. However, the above methods are laborious and time-consuming, which usually suffer from complicated and expensive instruments, multistep and complex sample preparation and skilled operators. Alternatively, electrochemical methods are good candidates for *in-situ* rapid monitoring and detection of TBBPA owing to their intrinsic advantages, which include relatively low-cost instrumentation, easy operation and high sensitivity. However, little endeavor has been devoted to fabricate the electrochemical sensor for TBBPA determination. At present, several indirect electrochemical methods have been reported for the determination of TBBPA based on the molecularly imprinted electrochemical sensors, and the [Fe (CN) _6_]^4−/3−^ was used as the indicator^29^,^30^,^31^. However, the studies about direct electrochemical detection of TBBPA are very limited^32^,^33^, and the sensitivity is relatively lower. Therefore, the development of new electrochemical sensors for TBBPA detection is given focus.

Past decades have witnessed remarkable progress on the nanotechnology, especially the application of the nanomaterial in the medical, biological and environmental analysis.

Since its discovery, multi-walled carbon nanotubes (MWCNTs) are considered as ideal candidate in the fabrication of electrochemical sensors^34^. MWCNTs can enhance the sensitivity of the electrochemical detection because of their attractive electronic, chemical and mechanical properties^35^,^36^,^37^,^38^,^39^. Moreover, carboxyl-functionalized multi-walled carbon nanotubes (MWCNTs-COOH) have better dispersion and stability compared with MWCNTs^40^. Furthermore, many unique properties of MWCNTs can be realized after integrated into more complex assemblies to improve its versatility.

In the meantime, Fe_3_O_4_ is a type of magnetic nanoparticle that is environmentally friendly, low cost, easy to prepare and possesses excellent water solubility^41^. In addition, Fe_3_O_4_ exhibits good electrical properties owing to the electron transfer between Fe^2+^ and Fe^3+^, this could enhance the electrode conductivity and facilitate electron transfer^42^,^43^. Therefore, coupling MWCNT-COOH with Fe_3_O_4_ as the sensing medium can improve the electron transfer and enhance the analytical sensitivity of electrochemical sensors efficiently. The resulting MWCNTs-Fe_3_O_4_ nanocomposite brings new capabilities for electrochemical sensing due to the synergetic effect between Fe_3_O_4_ and MWCNT.

Surfactants, which contain hydrophilic and hydrophobic groups, can change the properties of the electrode/solution interface and subsequently influence the electrochemical processes^44^. The modification of the surfactants on the electrode surface might significantly facilitate the electron transfer, greatly enhance the peak current and change the redox potentials^45^,^46^. Trimethyloctadecylammonium bromide (TOAB), a type of cationic surfactants, may play as an important role in enriching the TBBPA with its long alkyl chain. Moreover, the modification of the electrode surface by TOAB may increase the electron transfer between the electrode surface and analyte, which may improve the detection limit.

To the best of our knowledge, limited studies have reported the determination of TBBPA using the MWCNTs-Fe_3_O_4_ composite film and TOAB. This study described the preparation and characterization of the GCE modified with the composite of TOAB/MWCNTs-Fe_3_O_4_. The new electrochemical sensor showed the integrated properties of three components and the improvement of the sensitivity and accuracy. Finally, the new sensor was utilized for the determination of TBBPA in environmental water samples successfully.

## Results and Discussion

### The characterization of the as-prepared MWCNTs- Fe_3_O_4_ nannocomposite was investigated

**Figure 1:**
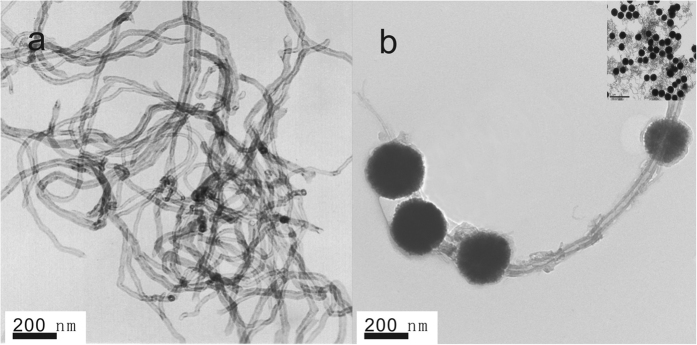
TEM images of (**a**) MWCNTs and (**b**) MWNTs-Fe_3_O_4_. Inset: The TEM image of MWNTs-Fe_3_O_4_ with 1 μm scale.

The Transmission electron microscopy (TEM) was used to characterize the microstructures of MWCNTs-COOH and the MWCNTs-Fe_3_O_4_ nanocomposite (Fig. 1). The Fig. 1a showed the morphology of MWCNTs-COOH. And Fig. 1b revealed that the Fe_3_O_4_ nanoparticles with diameter of 200 nm were coated on the MWCNTs-COOH, which confirmed the formation of the MWCNTs-Fe_3_O_4_ nanocomposite instead of the physical mixture of the two components.

**Figure 2:**
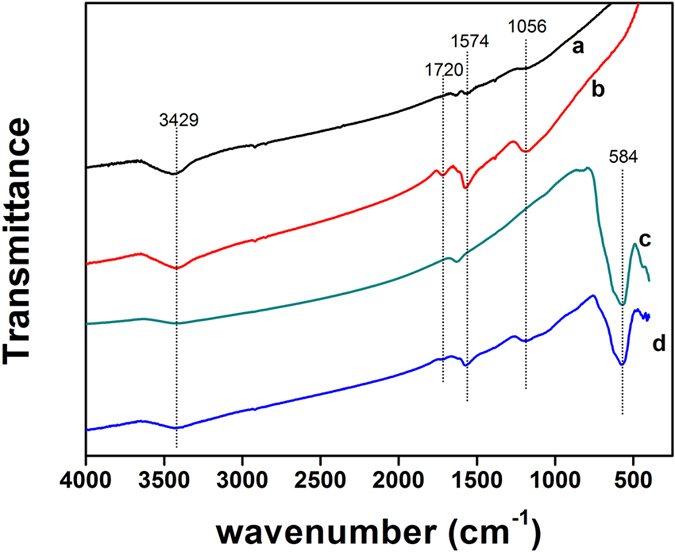
FT-IR spectra of MWCNTs (curve a), MWCNTs-COOH (curve b), Fe_3_O_4_ nanoparticles (curve c) and MWNTs-Fe_3_O_4_ (curve d).

The Fourier transform infrared (FT-IR) spectra provide information regarding the changes in surface functional group of the samples. The FT-IR spectra of the MWCNTs, MWCNTs-COOH, Fe_3_O_4_ nanoparticles and MWCNTs-Fe_3_O_4_ were given in Fig. 2. The peak at 3429 cm^−1^ arose from the hydroxyl group (–OH) stretching vibration of adsorbed water. Compared with MWCNTs, several significant changes in the peak intensity of the oxidized MWCNTs spectrum were observed. The FT-IR spectra of oxidized MWCNTs showed a peak intensity at1720cm^−1^ (C=O stretching) and 1056 cm^−1^ (C–O stretching). The band at 1574 cm^−1^ was the characteristic graphite structure peak, which was assigned to the C=C groups of the MWCNTs^47^. The FT-IR spectra of Fe_3_O_4_ nanoparticles and MWCNTs-Fe_3_O_4_ both contained bands at 584 cm^−1^ assigned to the Fe–O–Fe stretching and bending modes^48^, which indicated the attachment of Fe_3_O_4_ on the surface of MWCNTs. Moreover, the FT-IR spectra of MWCNTs allied with Fe_3_O_4_ showed the reduction in C=O stretching of MWCNTs-Fe_3_O_4_ spectrum at 1720 cm^−1^, which designated the utilization of –COOH of MWCNTs by co-precipitation with Fe_3_O_4_ particles. This may also indicate the successful preparation of the MWCNTs-Fe_3_O_4_ composite.

**Figure 3:**
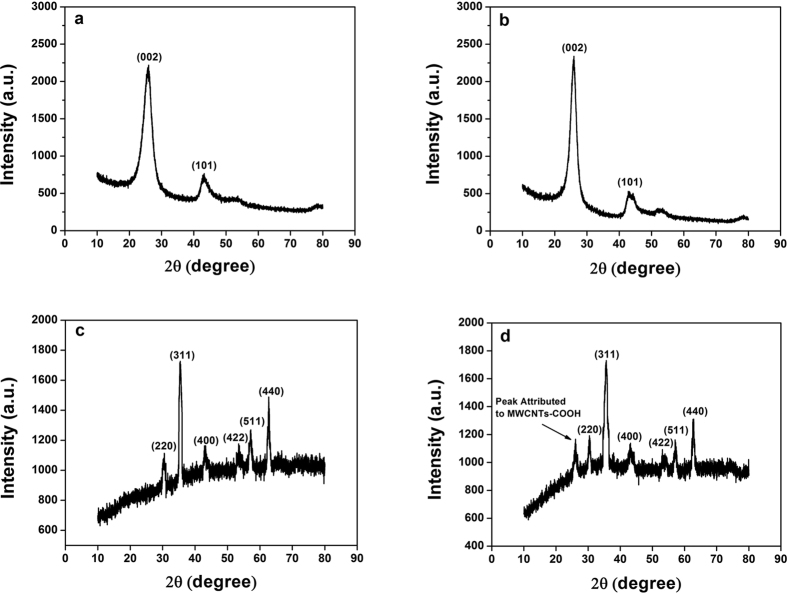
XRD patterns of (**a**) MWCNTs, (**b**) MWCNT-COOH, (**c**) Fe_3_O_4_ and (**d**) MWCNTs-Fe_3_O_4_.

The X-ray diffraction (XRD) patterns indicated that the crystal structures of the materials. As shown in Fig. 3a, the diffraction peaks at 2θ = 26.1° was identified as the (002) plane of the hexagonal graphite structure, which is consistent with previous reports^49^. The XRD pattern of MWCNTs-COOH (Fig. 3b) was similar to that of the MWCNTs. However, for MWCNTs-COOH, the diffraction peaks had higher intensities, and the crystallization peaks were more dominant. One possible reason is that the acid treatment of the MWCNTs advanced the removal of the amorphous carbon, carbon nanoparticles and metal particles. And as presented in Fig. 3c and d, the diffraction peaks of Fe_3_O_4_ at 2θ values of 30.58°, 35.56°, 43.32°, 53.75°, 57.27° and 62.77° were identified as the (220), (311), (400), (422), (511) and (440) crystal planes in Fe_3_O_4_ (JCPDS No. 65–3107), respectively, which agreed with the reported values^50^,^51^. A diffraction peak at 2θ = 26.1° also appeared in the XRD pattern of MWCNTs-Fe_3_O_4_ composite (Fig. 3d). The MWCNTs showed a lower intensity is probably ascribed to the reason that the Fe_3_O_4_ grafted on the surface of MWCNTs weakened the diffraction peak at 26° of MWCNTs. The XRD results indicated that MWCNTs-Fe_3_O_4_ nanocomposites were successfully synthesized by a facile hydrothermal method. No crystalline impurities were detected.

### The comparative studies on the electrochemical behavior of different modified electrode were also investigated

**Figure 4:**
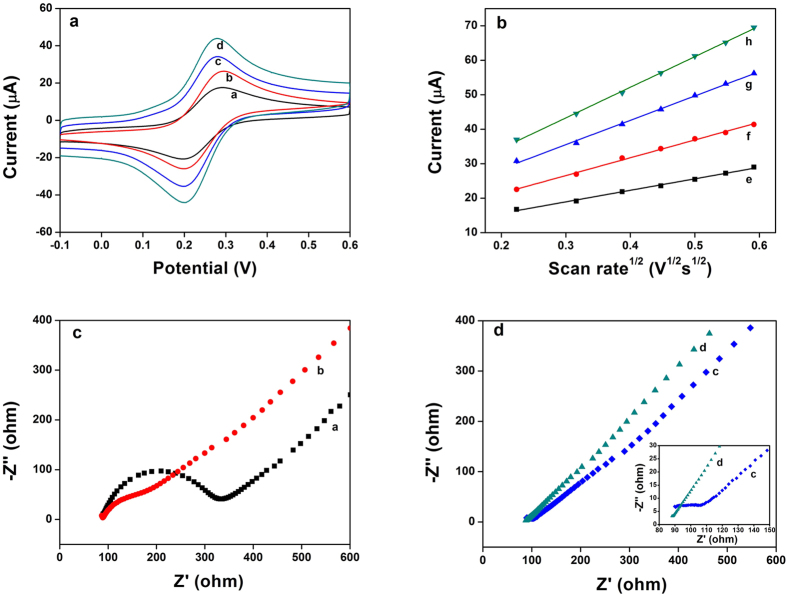
(**a**) Cyclic voltammograms of GCE (curve a), MWCNTs/GCE (curve b), MWCNTs-Fe_3_O_4_/GCE (curve c) and TOAB/MWCNTs-Fe_3_O_4_/GCE (curve d) in 0.1 M KCl solution containing 1 mM [Fe(CN)6]^3−/4−^ at a scan rate of 100mVs^−1^. (**b**) The plot of anodic peak current of [Fe(CN)6]^3−/4−^ (1 mM) vs. square root of scan rate for: GCE(curve e), MWCNTs/GCE (curve f), MWCNTs-Fe_3_O_4_/GCE (curve g)and TOAB/MWCNTs-Fe_3_O_4_/GCE (curve h). (**c,d**) Electrochemical impedance spectroscopy of GCE (curve a), MWCNTs/GCE (curve b), MWCNTs-Fe_3_O_4_/GCE (curve c) and TOAB/MWCNTs-Fe_3_O_4_/GCE (curve d) in 0.10 M KCl containing 5 mM [Fe(CN)6]^3−/4−^, frequency range: 0.1 kHz to 10 kHz with amplitude of 5 mV. Inset: Electrochemical impedance spectroscopy of MWCNTs-Fe_3_O_4_/GCE (curve c) and TOAB/MWCNTs-Fe_3_O_4_/GCE (curve d) at the high-frequency region.

The cyclic voltammetry method (CV) is an effective and convenient technique for probing the feature of the developed sensor. To investigate the electrochemical behaviors for each assembly step, different modified electrodes were studied with a 0.1 M KCl solution containing 1.0 mM [Fe (CN) _6_]^4−/3−^ probe at scan rate of 100 mV s^−1^. As seen in Fig. 4a, a pair of well-defined redox peaks of [Fe (CN) _6_]^4−/3−^ was observed on the surface of bare GCE. After the GCE was modified with MWCNTs, the peak current increased because MWCNTs could increase surface area and active sites for electron transfer. With the introduction of Fe_3_O_4_ nanoparticles, the peak current increased significantly, which suggested the addition of Fe_3_O_4_ enhanced furtherly effective area and active sites for electron transfer. Finally, after modification with TOAB, the electrochemical behavior of the [Fe (CN) _6_]^4−/3−^ on the TOAB/MWCNTs-Fe_3_O_4_/GCE was obviously improved, which indicated that the TOAB/MWCNTs-Fe_3_O_4_/GCE exhibited a faster electron transfer rate and larger electroactive surface area compared with the former electrodes.

The effective surface areas of the GCE, MWCNTs/GCE, MWCNTs-Fe_3_O_4_/GCE and TOAB/MWCNTs-Fe_3_O_4_/GCE were estimated by [Fe (CN) _6_]^4−/3−^ redox system with different scan rate. For a reversible process, the Randles–Sevcik equation^52^ is as follows:


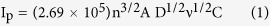


where Ip is the anodic peak current, ν is the scan rate, n is counted as the number of electron transferred in the redox reaction, C is the concentration of [Fe (CN) _6_]^4−/3−^, A is the effective surface area of electrode, D represents the diffusion coefficient of the [Fe (CN) _6_]^4−/3−^ probe. For 1 mM [Fe (CN) _6_]^4−/3−^ in an aqueous solution, n = 1, D = 6.7 × 10^–6^ cm^2^ s^−1^. Thus, as shown in Fig. 4b, from the slope of the anodic peak current (Ipa) versus square root of scan rate (v^1/2^) relation, the effective surface area of bare GCE, MWCNTs/GCE, MWCNTs-Fe_3_O_4_/GCE and TOAB/MWCNTs-Fe_3_O_4_/GCE were calculated to be 0.048 cm^2^, 0.076 cm^2^, 0.101 cm^2^ and 0.127 cm^2^, respectively. The effective surface area of TOAB/MWCNTs-Fe_3_O_4_/GCE is nearly 2.65 times as large as the bare GCE, which indicated that the effective surface area was remarkably increased in the presence of MWCNTs-Fe_3_O_4_ and the TOAB.

Electrochemical impedance spectroscopy (EIS) is an effective technique for probing the features of modified electrodes. The impedance spectra include a semicircle portion and a linear portion. The linear part at lower frequencies represents the diffusion process, whereas the semicircle portion at higher frequencies corresponds to the electron-transfer limit process, and the diameter of the semicircle indicates the electron transfer resistance (Ret)^53^. The stepwise construction process of the sensor was characterized by EIS, and the EIS patterns of different electrodes in a solution of 0.1 M KCl containing 5 mM [Fe (CN) _6_]^4−/3−^ was recorded. As shown in Fig. 4c, a big well-defined semicircle at higher frequencies obtained for the bare GCE (curve a), which indicated a huge interface electron transfer resistance. When MWCNTs were modified on the GCE, a small semicircle in the Nyquist plot was obtained (curve b), which indicated that interface electron transfer resistance decreased obviously. For the MWCNTs-Fe_3_O_4_ modified electrode (Fig. 4d, curve c), a little smaller semicircle in the Nyquist plot was obtained compared with curve b, which showed a smaller interface electron transfer resistance. And this phenomenon could be ascribed to cooperative action between MWCNTs and Fe_3_O_4_ nanoparticles. After introduction of TOAB, the electrode modified with the TOAB/MWCNTs-Fe_3_O_4_ showed the smallest semicircle, which indicated that the conductivity of the TOAB/MWCNTs-Fe_3_O_4_ modified electrode was better than the other electrodes. This is good for the electrochemical sensing. These results agreed with the performance of the above-mentioned cyclic voltammograms.

The enrichment effect of different modified electrodes was also studied using chronocoulometry method. The curves of charge (Q)-time (t) in pH 7.0 phosphate buffer in the absence and presence of 100.0 nM TBBPA were individually recorded. Then, the data of Q-t curves were converted to the Q-t^1/2^ plots, and two fitted curves were obtained (see Supplementary Fig. S1). According to the integrated Cottrell equation^54^:


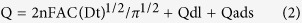


where Qdl is the double-layer charge, Qads is the Faradaic charge owing to the oxidation of adsorbed TBBPA, n is the electron transfer number, A is the electrode area, C is the concentration, D is the diffusion coefficient, and F is the Faraday constants. Therefore, the intercept value of Q-t^1/2^ plot in the blank phosphate buffer without TBBPA represented Qdl, and the intercept value in the presence of TBBPA is the summation of the Qdl and Qads. As illustrated in the Supplementary Fig. 1, the Qads of the TBBPA on different electrodes could be easily calculated, the values on GCE, MWCNTs/GCE, MWCNTs-Fe_3_O_4_/GCE and TOAB/MWCNTs-Fe_3_O_4_/GCE were 0.322, 1.117, 1.521 and 3.516 μC, respectively. The improvements of Qads demonstrated a significant enrichment effect toward TBBPA in the presence of MWCNTs-Fe_3_O_4_ and the TOAB. Moreover, after the modification of the TOAB onto the electrode, the Qads value of TBBPA increased obviously, which indicated that the accumulation ability of TBBPA was improved obviously. This phenomenon may be attributed to the strong intermolecular force and hydrophobic interaction between the TOAB and TBBPA. It has been reported that the surfactants can enrich phenol compounds by its hydrophobic tails^55^. The TOAB, with three C18-alkyl chains, may play an important role in the enrichment of TBBPA. The long alkyl chains might provide a hydrophobic micro-environment on the surface of the electrode^33^, which may enhance the absorption of the lipophlic chemical TBBPA onto the electrode surface.

Thus, the TOAB could facilitate the electron transfer, enlarge the effective surface area of sensor, and enrich the TBBPA with its long alkyl chains effectively. The results of chronocoulometry, along with the results of EIS and cyclic voltammograms, indicated that there may be a cooperative effect between each component in the stepwise construction process of the sensor.

### The synergetic signal amplification of MWCNTs-Fe_3_O_4_ nanocomposite hybrid and TOAB was studied

**Figure 5:**
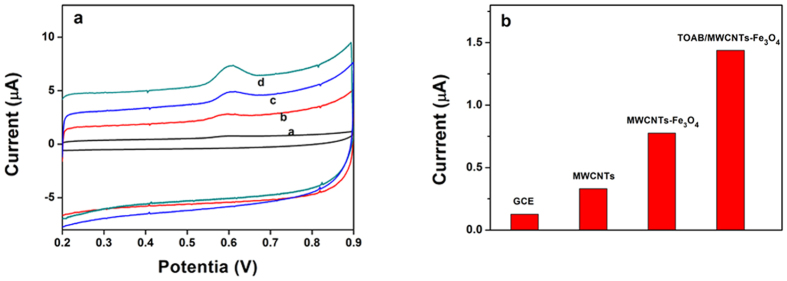
(**a**) Cyclic voltammograms of 100.0 nM TBBPA at the GCE (curve a), MWCNTs/GCE (curve b), MWCNTs-Fe_3_O_4_/GCE (curve c) and TOAB/MWCNTs-Fe_3_O_4_/GCE (curve d) in 0.1 M phosphate buffer, scan rate: 100 mV s^−1^. (**b**) Oxidation peak current of TBBPA on different modified GCEs surface.

The oxidation behaviors of TBBPA on different modified electrodes were studied to deeply discuss their signal enhancement effects. The Fig. 5a records the detailed CV curves and Fig. 5b illustrates the variation of oxidation peak current (Ip) in the solution of pH 7.0 PBS containing 100.0 nM TBBPA. As shown in the Fig. 5a, only an oxidation peak was observed during the successive cyclic sweep from 0.2 to 0.9 V. Thus, the oxidation of TBBPA was totally irreversible on the surface of different modified electrodes. It could be seen in the Fig. 5, on the surface of bare GCE, a weak oxidation peak appeared at 0.59 V with the peak current of 0.12 μA owing to the poor response to TBBPA. When using MWCNTs/GCE, the oxidation peak currents increased considerably and the peak current became 0.33 μA, which suggested that the prepared MWCNTs displayed remarkable signal enhancement on TBBPA oxidation. The oxidation peak currents of TBBPA was further enhanced on the surface of MWCNTs-Fe_3_O_4_ hybrid film, and the peak current further increased to 0.77 μA, the value of oxidation peak currents are increased by 6.4-fold in comparison with those on bare GCE. And, the oxidation responses of TBBPA in the presence of TOAB were also examined. It should be noted here that the oxidation peak currents of TBBPA increased significantly after addition of TOAB. The peak current of TOAB/MWCNTs-Fe_3_O_4_ modified GCE was 1.43 μA, which was almost 12 times as large as the peak current of bare GCE. Such peak current enlargements testify the strong signal amplification of TOAB. From Fig. 5, we clearly found that the response signals of TBBPA are the highest on MWCNTs-Fe_3_O_4_ hybrid film surface with the assistance of TOAB.

Based on the results of electrochemical comparative studies and the chronocoulometry study, there are two main reasons may account for the mechanism of synergetic signal enhancement between each component. On the one hand, the effective surface area and the electron transfer rate of electrodes were improved by each modification step. Therefore, the electrochemical performance towards TBBPA may be improved in the construction process. On the other hand, the accumulation ability of different electrodes was also improved as demonstrated in the chronocoulometry study. The bare GCE showed a poor response to TBBPA, which may be attributed to the poor electrochemical performance and accumulation ability of bare GCE. When MWCNTs was modified onto the GCE, the electrochemical performance and accumulation ability were both improved. This may be ascribed to the higher electron transfer rate and good conductivity after the modification of MWCNTs. And the TBBPA could be absorbed on the nano-structure of MWCNTs. Consequently, the larger oxidation current peak of TBBPA was showed on the MWCNTs/GCE. After the Fe_3_O_4_ nanoparticles were introduced onto the MWCNTs, the MWCNTs-Fe_3_O_4_/GCE showed a further improvement of electrochemical performance and accumulation ability. Fe_3_O_4_ exhibits good electrical properties owing to the electron transfer between Fe^2+^ and Fe^3+^, this could enhance the electrode conductivity and facilitate electron transfer^43^. So, the electrochemical performance of MWCNTs-Fe_3_O_4_/GCE was further improved compared with MWCNTs/GCE. At the same time, the three-dimensional structure of MWCNTs-Fe_3_O_4_ hybrid could absorb more target molecule^56^. Thus, the signal enhancement effect could be observed. Finally, when TOAB was modified on the electrode, electrochemical performance and accumulation ability were greatly improved on the surface of the MWCNTs-Fe_3_O_4_/GCE. This could be explained by the reasons discussed above. Hence, the further signal enhancement was showed. To summarize, the system of MWCNTs-Fe_3_O_4_ hybrid and TOAB exhibited very strong synergetic signal amplification on the oxidation of TBBPA, which confirmed our original hypothesis, and the detection sensitivity could be greatly improved.

### The effects of different experimental conditions on the signal response were investigated

he effect of TOAB concentration on the oxidation response signal of TBBPA was also discussed. The Supplementary Figure. S2 shows that the oxidation peak currents of TBBPA increased greatly with TOAB concentration from 0 to 0.19 mM (75 mg L^−1^), and then gradually decreased with the increasing TOAB concentration. The notable peak current improvements manifest that the prepared surfactant films are more active for the direct oxidation of TBBPA. Herein, the concentration of TOAB was fixed at 0.19 mM for optimum sensitivity.

**Figure 6:**
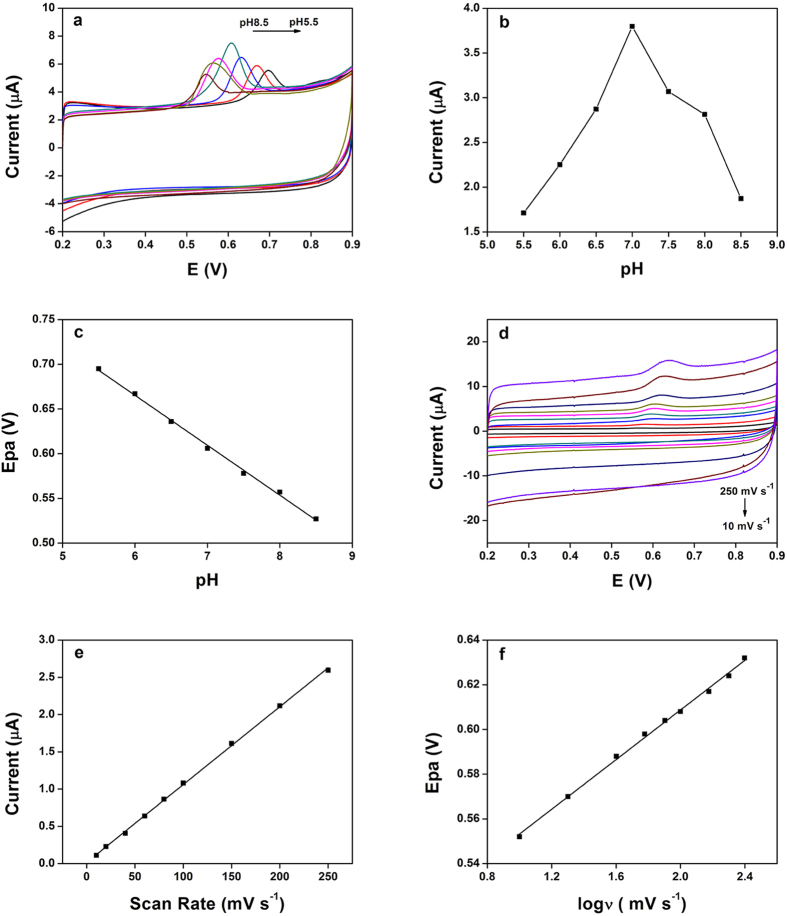
(**a**) CV curves of 500.0 nM TBBPA on TOAB/MWCNTs-Fe_3_O_4_/GCE in 0.1 M phosphate buffer with different pH values, scan rate: 100 mV s^−1^. (**b**) Effect of pH value on the oxidation peak current of 500.0 nM TBBPA. (**c**) The plot of anodic peak potential (Epa) of TBBPA versus pH values. (**d**) Cyclic voltammograms of 100.0 nM TBBPA on TOAB/MWCNTs-Fe_3_O_4_/GCE at different scan rates (10, 20, 40, 60, 80, 100, 150, 200 and 250 mV s^−1^). (**e**) Relationship between anodic peak current and potential scan rate. (**f**) Linear regression of the Epa versus the logarithm of scan rates.

The effect of pH value was also discussed. In most cases, the electrolyte pH is an important parameter in the electrochemical reaction. The influence of the solution pH on the electrocatalytic oxidation of TBBPA (500.0 nM) was investigated within the pH range of 5.5 to 8.5 phosphate buffer (Fig. 6a). As displayed in Fig. 6b, the oxidation peak currents of TBBPA on the TOAB/Fe_3_O_4_-MWNTs/GCE increased gradually when pH value improved from 5.5 to 7.0, and then decreased gradually with further improving pH value. Therefore, the pH 7.0 phosphate buffer solution was chosen for the subsequent analytical experiments to achieve higher response signal. The oxidation peak potential shifted negatively with the increase of pH value, which indicated that the protons were involved in the electrode reaction when pH increased from 5.5 to 8.5. Moreover, a good linear relationship can be established between anodic peak potential (Epa) and the solution pH. As shown in Fig. 6c, the regression equation can be expressed as Epa (V) = −0.0559 pH + 1.004 (R^2^ = 0.9979). The calculated slope value of 0.0559 V pH^−1^ is approximate close to the theoretical value of 0.0592 V pH^−1^ according to the Nernst equation, which indicates that the electron transfer is accompanied by an equal number of protons in the electrode reaction.

The effect of scan rate on the electrochemical behavior of TBBPA (100.0 nM) at the TOAB/Fe_3_O_4_-MWNTs/GCE was investigated by cyclic voltammetry. The Fig. 6d shows that, at scan rates from 10 to 250 mV s^−1^, the oxidation peak currents (Ipa) of TBBPA increase linearly with the scan rates. The linear equation of TBBPA is Ipa (μA) = 0.0104ν (mV s^−1^) + 0.0166 (R^2^ = 0.9994) (Fig. 6e). The results indicate that the process was predominantly adsorption-controlled process. In addition, there was a linear relation between anodic peak potential (Epa) and logarithm of scan rate (logν), which corresponds to the following equation: Epa (V) = 0.0555logν (mV s^−1^) + 0.4977 (R^2^ = 0.9976) (Fig. 6f). In the case of an adsorption-controlled and irreversible electrode process, Laviron mentioned that the slope of the line is equal to 2.3RT/(αnF)^57^. Moreover, α was assumed to be 0.5 in totally irreversible electrode process^58^. Therefore, based on the value of the equation above, the electron transfer number (n) could be calculated as 2 in this study.

The electrochemical oxidation of TBBPA on TOAB/MWCNTs-Fe_3_O_4_/GCE was suggested to be a two-electron and two-proton process given that the number of electron and proton involved in the oxidation process of TBBPA was equal as demonstrated in the pH-dependent electrochemical response. The electro-oxidation mechanism of TBBPA could be illustrated in Supplementary Fig. S3.

Fixing the accumulation potential and accumulation time was significant when adsorption studies were intended. Both conditions could affect the amount of adsorption of TBBPA at the electrode. Thus, the effect of accumulation potential and time on the CV signal was studied. The concentration of TBBPA was 100.0 nM. A little change in the peak current was observed when accumulation potential varied from −0.30 to +0.30 V. However, comparison of the obtained peak current for the open-circuit accumulation with those for polarization accumulation did not show significant difference. Therefore, the open circuit accumulation was applied. The influence of accumulation time ranges from 0 to 10 min on the oxidation of TBBPA at TOAB/MWCNTs-Fe_3_O_4_/GCE was also investigated (see Supplementary Fig. S4). The peak current increased gradually as the accumulation time increased from 0 to 6 min. However, the peak current tends to be almost stable with the further increase of accumulation time beyond 6 min. Therefore, the optimal accumulation time of 6 min was chosen in further investigations.

### The electrochemical Determination of TBBPA was studied

**Figure 7:**
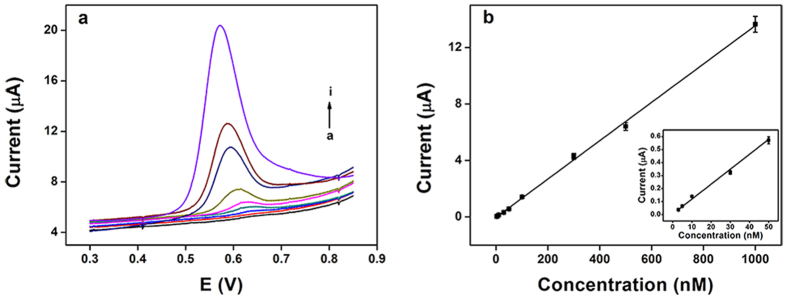
(**a**) DPV for different concentrations of TBBPA (a-i: 3, 5, 10, 30, 50, 100, 300, 500, 1000 nM) on the TOAB/MWCNTs-Fe_3_O_4_/GCE in 0.1 M PBS with pH 7.0. (**b**) Calibration curve of TBBPA on the TOAB/MWCNTs-Fe_3_O_4_/GCE electrode under the optimized conditions. Error bar represents the standard deviation of five measurements. Inset: The amplification of the calibration curve from 3 nM to 50 nM.

Differential pulse voltammetry (DPV) technology was used for quantitative detection of TBBPA because of the sensitivity of DPV was higher than the CV. Under the optimal conditions, DPV was performed to investigate the relationship between the peak current and the concentration of TBBPA. The pulse amplitude was 50 mV, the pulse width was 50 ms, and the scan rate was 40 mV s^−1^. As shown in Fig. 7a and b, the oxidation peak current of TBPPA increased linearly with the concentration in the range from 3.0 to 1000.0 nM. The linear regression equation was calculated as: Ipa (μA) = 0.0136 C (nM) − 0.022 (R^2^ = 0.9988), and the limit of detection was estimated to be 0.73 nM based on three signal-to-noise ratio (S/N).

The repeatability of one TOAB/MWCNTs-Fe_3_O_4_/GCE was examined by the detection of 100.0 nM TBBPA for five successive determinations. The relative standard deviation (RSD) is 2.85%, which shows that the excellent repeatability of the modified electrode for TBBPA detection was obtained. Fabrication reproducibility was also estimated with five different electrodes, which were fabricated independently by the same procedure. The RSD is 4.67% for the peak current measured in 100.0 nM TBBPA, which demonstrates the reliability of the fabrication procedure. In addition, the fabricated electrode retained 93.04% of its initial response after stored in the refrigerator at 4 °C for one week, which indicates that the good long-term stability of the TOAB/MWCNTs-Fe_3_O_4_/GCE can be achieved.

The influence of potentially interfering substances on the determination of analyte compound was investigated to evaluate the selectivity of the method for the determination of TBBPA. The tolerance limit for interfering species was considered as the maximum concentration that gave a relative error less than ± 5.0% at a concentration of 100.0 nM of TBBPA. Table S1 presents that the inorganic species K^+^, Na^+^, NH^4+^, Ca^2+^, Mn^2+^, Cd^2+^, Zn^2+^, Cu^2+^, Al^3+^, Fe^2+^, Fe^3+^, Cl^−^, Ac^−^, SO4^2−^, and PO4^3−^ in a 500-fold concentrations almost had no influence on the detection of 100.0 nmol L^−1^ TBPPA with deviations below 5% (see in the Supplementary Table S1). Moreover, 100-fold concentration of some organic substances and organic phenolic compounds such as glucose, p-aminophenol, 4-nitrophenol, 3-aminophenol, o-nitrophenol, m-nitrophenol, p-nitrophenol, catechol, hydroquinone, phenol, nonyl phenol, showed no influence on the electrochemical signal with deviations below 5%.

Although, bisphenol A (BPA), 4, 4′-Dihydroxydiphenylmethane (BPF), 4,4-Dihydroxydiphenyl sulphone (BPS) hexafluorobisphenol A (BPAF), 2, 2′,6, 6′- tetrabromobisphenol A diallyl ether (TBBDE), tetrabromobisphenol A bis (dibromopropyl ether) (TBBME), 4, 4′-Sulphonylbis (2, 6-dibromophenol) (TBBPS), tetrachlorobisphenol A (TCBPA), showed interference effect on the determination of TBBPA in different levels owing to the similar structure to TBBPA, the signal changes were all less than 5%, thereby indicating the selectivity of the prepared sensor was acceptable. The comparison with other reported methods shown in Supplementary Table S2 reveals that the newly developed TOAB/MWCNTs-Fe_3_O_4_/GCE based sensor is sensitive and competitive.

### For the further evaluation of the applicability of the method, the proposed sensor was applied to detect TBBPA in water samples collected from the environment

And, to verify the performance of the proposed method, HPLC method was used to determine TBBPA in real samples at the same time. The standard addition method was used for calculating the TBBPA concentrations given that TBBPA had not been detected from original water samples. The results obtained are shown in Table 1. The recovery ranged from 95.5% to 106.5%, and relative standard derivation (RSD, n = 5) was below 5.0%. These results indicated that the relative standard derivations (RSDs) and the recovery rates were acceptable. Furthermore, the results obtained by the proposed electrochemical method were in a good agreement with the HPLC method. Thus, the prepared sensor could be applied reliably and effectively for the determination of TBBPA in water samples.

## Conclusions

In summary, a novel electrochemical sensor was constructed to determine TBBPA in the water samples. After hybridization with Fe_3_O_4_ nanoparticles, the obtained MWCNTs-Fe_3_O_4_ hybrid exhibited stronger signal enhancement on TBBPA oxidation, compared with MWCNTs. The addition of TOAB further enhanced the oxidation signal of TBBPA and displayed remarkable synergetic signal amplification effects. This new sensing method exhibited excellent sensitivity and stability with the detection limit as low as 0.73 nM. Finally, the new sensing method could be successfully applied to detect TBBPA in water samples with satisfactory results.

## Methods

### Reagents and apparatus

Tetrabromobisphenol A (TBBPA, 99%) and Trimethyloctadecylammonium bromide (TOAB) were purchased from Sigma-Aldrich. The 0.01 mol L^−1^ of stock solution of TBBPA was prepared with methanol and stored at 4 °C. The multi-walled carbon nanotubes (MWNTs, purity > 95%) was obtained from the Nanjing XFNano Advanced Materials Supplier (Nanjing, China). All other reagents and solvents were of analytical grade and used without further purification. Ultrapure water was produced from a Milli-Q purification system (Millipore, Germany) and used throughout the experiments. The phosphate buffer solutions (PBS) were prepared in the pH range 5.5 to 8.5 and were used as supporting electrolytes.

Electrochemical measurements were performed with a CHI660C electrochemical workstation (CH Instruments, Shanghai, China). A conventional three-electrode system was used, containing a bare or modified glassy carbon electrode as the working electrode (Φ = 3 mm), a saturated calomel electrode (SCE) as the reference electrode and a platinum wire as the counter electrode. X-ray diffraction (XRD) pat-terns were measured using a X’Pert PRO diffractometer (Panalytical Company, Netherlands). Transmission electron microscopy (TEM) images were recorded with a Tecnai G220U-Twin microscope (FEI Com-pany, Netherlands). The Fourier transform infrared (FTIR) spectra were recorded with a VERTEX 70 spectrometer (Bruker, Germany). The high-performance liquid chromatograph (HPLC) was performed on Agilent 1200 series with a UV-vis detector (Agilent Technologies, United States). The analytical column used was Waters Sunfire C18 column (150 × 4.6 mm, 5 mm). The mobile phase was methanol-water (85/15, v/v) and the flow rate was 1.0 mL min^−1^. The injection volume was 20 μL and the UV spectra was recorded at 212 nm. All measurements were performed at ambient temperature.

### Synthesis of Fe_3_O_4_/MWNTs-COOH nanocomposite

Initially, chemical pretreatment is a simple and effective way to introduce oxygen groups (COOH, C=O and OH) onto the MWCNTs surface to improve its properties^59^. The MWCNTs-COOH was prepared according to a method reported by Zhang^60^. An amount of 500 mg of MWCNTs was added to a 60 mL solution of H_2_SO_4_–HNO_3_ (3 : 1, v/v raio) and the mixture was ultrasonicated for 15 min. Then, the mixture was stirred at 85 °C for 12 h. After cooling to room temperature, the product was isolated by filtration through a 0.22 mm polycarbonate membrane and washed with water several times until the pH of the filtrate was neutral. Finally, the resulting MWCNTs-COOH was dried under vacuum at 40 °C for 12 h.

NaAc (3.6 g) and a suitable amount of MWCNTs were then added into the homogeneous solution of FeCl_3_ (0.81 g) and dissolved in ethylene glycol (40 mL). Ultrasonic for 30 min was employed to disperse the mixture and the mixture was then transferred to a teflon-lined stainless-steel autoclave. The autoclave was heated to and maintained at 200 °C for 8 h, and allowed to cool to room temperature. Finally, the black products were obtained and washed several times with water and ethanol and then dried at 60 °C.

### Preparation of working electrodes

First, in order to obtain the stable MWCNTs- Fe_3_O_4_ suspension, 10.0 mg of MWCNTs- Fe_3_O_4_ composite was exactly weighed and added into 10.0 mL dimethylformamide (DMF). After an ultrasonic treatment for 60 min, a stable and black suspension with concentration of 1 mg mL^−1^ was achieved. The solutions of TOAB with concentration of 1.0 mg mL^−1^ was prepared using ethanol, and then diluted to different concentrations for the subsequent modification.

Prior to the preparation of the modified electrode, the bare glassy carbon electrode (GCE) was polished to obtain a mirror-like surface with 0.3 and 0.05 μm alumina slurry, respectively, and then sonicated in alcohol and ultrapure water successively to remove any residue. After drying in the air at room temperature, the clean GCE was modified with a certain amount of the MWCNTs-Fe_3_O_4_ suspension and dried under an infrared lamp. Afterwards, 2 μL of the diluted TOAB solution was dropped onto GCE surface, and kept at room temperature to evaporate ethanol completely. The prepared electrode was denoted as TOAB/MWCNTs-Fe_3_O_4_/GCE.

For the comparison, MWCNTs/GCE, MWCNTs- Fe_3_O_4_/GCE and TOAB/MWCNTs-Fe_3_O_4_/GCE were prepared with the similar procedure.

### Water sample preparation

Water sample was collected from the East Lake and Yangtze River (Wuhan, China) and detected using the presented method and HPLC method, respectively. The sample was filtered by 0.45 μm membrane prior to the electrochemical detection. The filtered water sample was then mixed with NaHPO_4_ and Na_2_PO_4_ for preparing 0.1 M phosphate buffer. The pH value was adjusted to 7.0. For HPLC method, the water sample was filtered by 0.45 μm and 0.22 μm membranes successively and then the pH value was adjusted to 7.0. The sample was then mixed with methanol (1: 1, v/v). All prepared samples were stored in refrigerator at 4 °C before analysis.

## Additional Information

**How to cite this article**: Zhou, F. *et al*. Synergetic signal amplification of multi-walled carbon nanotubes-Fe_3_O_4_ hybrid and trimethyloctadecylammonium bromide as a highly sensitive detection platform for tetrabromobisphenol A. *Sci. Rep.* **6**, 38000; doi: 10.1038/srep38000 (2016).

**Publisher's note:** Springer Nature remains neutral with regard to jurisdictional claims in published maps and institutional affiliations.

## Supplementary Material

Supplementary Information

## Figures and Tables

**Table 1:** Determination of TBBPA in real samples by the developed electrochemical method (n = 5) and HPLC method (n = 5).

Sample	Added (μmol L^-1^)	Electrochemical Method			HPLC Method		
		Found (μmol L^−1^)	Recovery (%)	RSD (%)	Found (μmol L^−1^)	Recovery (%)	RSD (%)
1	0.01	0.0096	96.3	4.1	0.0098	98.3	4.8
	0.10	0.1035	103.5	3.2	0.0955	95.5	3.6
	1.00	1.0471	104.7	2.1	0.9884	98.8	3.7
2	0.01	0.0097	97.3	3.8	0.0105	105.7	2.9
	0.1	0.0986	98.6	4.2	0.0977	97.7	3.4
	1.0	1.0211	102.1	3.4	0.9953	99.5	1.7
3	0.01	0.0103	103.1	3.8	0.0098	98.1	3.5
	0.1	0.1065	106.5	3.4	0.0964	96.4	1.6
	1.0	1.0221	102.2	2. 3	0.9919	99.19	2.1
