# Supplementary material for: Synergetic signal amplification of multi-walled carbon nanotubes-Fe3O4 hybrid and trimethyloctadecylammonium bromide as a highly sensitive detection platform for tetrabromobisphenol A
Source: Sci Rep. 2016 Nov 29;6:38000. doi: 10.1038/srep38000 (PMC5126559; doi:10.1038/srep38000)
Supplement: Supplementary Information [file srep38000-s1.pdf]

Supplementary information:

Synergetic signal amplification of multiwalled carbon nanotubes-Fe_3O_4 hybrid and trimethyloctadecylammonium bromide as a highly sensitive detection platform for tetrabromobisphenol A

Feng Zhou[a], Yue Wang[a], Wei Wu[a], Tao Jing[a], Surong Mei[a], Yikai Zhou[a]*

[a] State Key Laboratory of Environment Health (Incubation), Key Laboratory of Environment and Health, Ministry of Education, Key Laboratory of Environment and Health (Wuhan), Ministry of Environmental Protection, School of Public Health, Tongji Medical College, Huazhong University of Science and Technology, #13 Hangkong Road, Wuhan, Hubei 430030, China

[b] School of Laboratory Medicine, Hubei University of Chinese Medicine, #1 Hangjia Lake West Road, Wuhan, Hubei 430030, China

*** Corresponding author:**

Yikai Zhou

Tel.: +86(27)-83657849

Fax: +86(27)-83657765

E-mail: zhouyk@mails.tjmu.edu.cn

Address: School of Public Health, Tongji Medical College, Huazhong University of Science and Technology, #13 Hangkong Road, Wuhan, Hubei, 430030, China

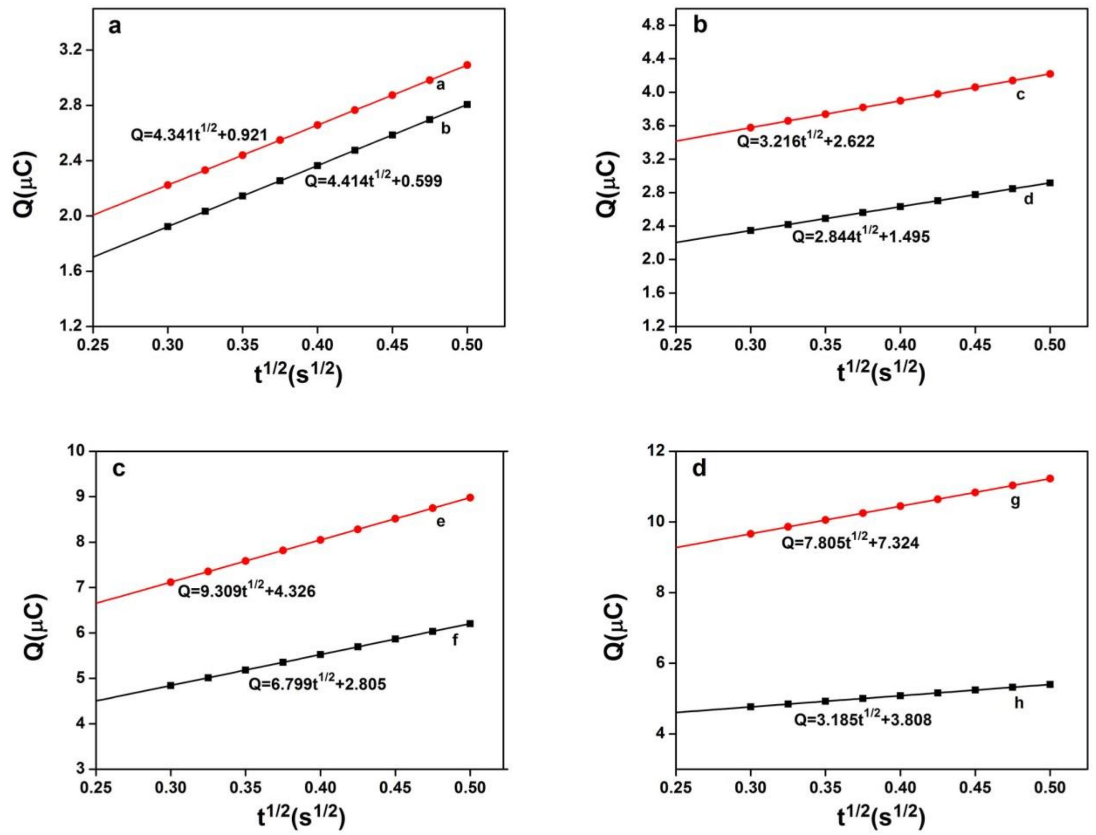

Figure S1. (a) Q-$t^{1/2}$ plots on GCE, (b) MWCNTs/GCE, (c) MWCNTs-Fe_3O_4/GCE and (d) TOAB/MWCNTs-Fe_3O_4/GCE in blank pH 7.0 PBS buffer (curve a, c, e and g) and in the presence of 100.0 nM TBBPA (curve b, d, f and h). Potential step: 0.3 to 0.8 V, plus width: 0.25 s.

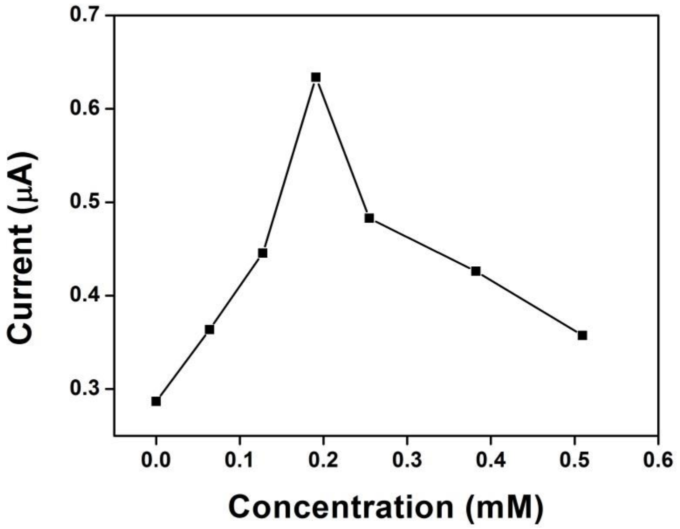

Figure S2. Influences of TOAB concentration on the oxidation peak currents of 100.0 nM TBBPA.

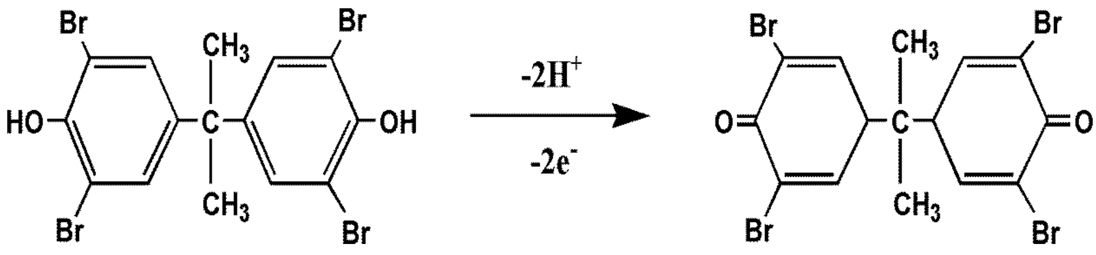

Figure S3. Schematic illustration of TBBPA electro-oxidation mechanism on the TOAB/MWNTs-Fe_3O_4/GCE.

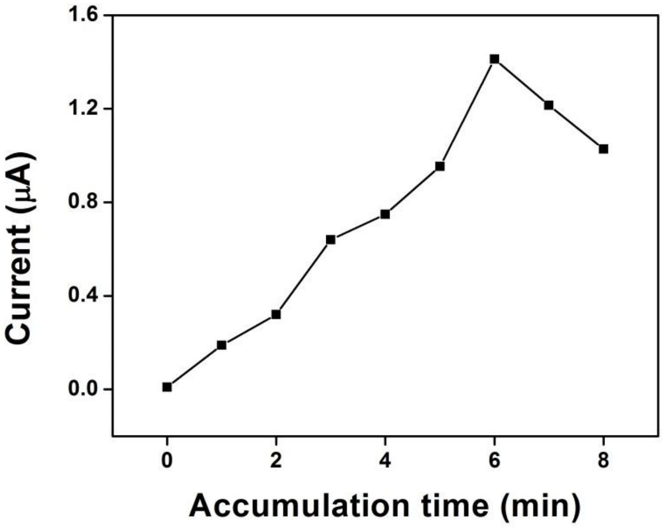

Figure S4. Influences of accumulation time on the oxidation peak currents of 100.0 nM TBBPA.

Table S1. Interference study for the determination of 0.1 μM TBBPA under the optimized condition

Interference	Tolerance limit (μM)
K^{+}, Na^{+}, NH^{4+}, Ca^{2+}, Mn^{2+}, Cd^{2+}, Zn^{2+}, Cu^{2+}, Al^{3+}, Fe^{2+}, Fe^{3+}, Cl^{-}, Ac^{-}, $SO4^{2-}$, $PO4^{3-}$	50
glucose, p-aminophenol, 4-nitrophenol, 3-aminophenol, o-nitrophenol, m-nitrophenol, p-nitrophenol, catechol, hydroquinone, phenol, nonyl phenol	10
BPAF, BPF, BPS, TBBDE, TBBME , TCBPA,	1
BPA, TBBPS	0.5

Table S2.The comparison of different methods for TBBPA detection.

Method	Linear range (nM)	Detection limit (nM)	Ref
HPLC	9.20–920.81	0.239	21
HPLC-MS	0.02–0.36	0.023	22
GC-MS	9.20–552.48	0.165	24
ELISA	0.11–10.84	0.05	27
MIP/GP/NBD/GCE	0.50–4.5	0.23	29
MIP/Ni/GP/GCE	0.5–10000	0.13	30
MIP/GP/CNTs/GCE	0.01–10	0.0037	31
AB/GCE	18.42 –644.56	11.12	32
CTAB /NG-TPA/GCE	10–1000	9	33
TOAB/MWCNTs-Fe_3O_4/GCE	3–1000	0.73	This work
